# The parasitophorous vacuole membrane of *Toxoplasma gondii*: A custom structural framing with smart interior plumbing for resource allocation

**DOI:** 10.1371/journal.ppat.1014473

**Published:** 2026-08-05

**Authors:** Julia D. Romano, Shahbaz M. Khan, Isabelle Coppens

**Affiliations:** Department of Molecular Microbiology and Immunology, Johns Hopkins School of Public Health, Baltimore, Maryland, United States of America; University of Wisconsin Medical School, UNITED STATES OF AMERICA

## Abstract

In essence, the parasitophorous vacuole (PV) of *Toxoplasma* is a “Do-It-Yourself” renovation project led by the parasite after invasion of a guest house (the host cell) to install itself surreptitiously in a private and custom-built room, reinforced by drywall (the PV membrane). The function of the PV membrane (PVM) is to keep away the host security guards (immune response, lysosomes) from the parasite while allowing necessary and controlled access to host resources. Inside the PV, *Toxoplasma* relies on a crew of creative interior designers (the GRA proteins or GRA) to remodel and transform the space into an efficient living and breeding suite. Some GRA set up specialized delivery systems in the PVM (pores) to siphon off groceries (nutrients) from the host cytosol. Other GRA build a complex network of tubules that attach to the PVM (intravacuolar network) to trap large grocery stores (host organelles) inside the PV, ensuring the massive supply of selective food (lipids). In a parallel circuit, specific GRA recruit host proteins at the PVM that shape this membrane and mediate scission (host ESCRT) to engulf more host resources. In addition to this interior layout, *Toxoplasma* establishes a plumbing system made of PVM tubules that pervade the host cell, which maximizes the surface area for nutrient uptake, and lasso host organelles (lipid droplets, ER) from the surroundings. Finally, the PV acts as a magnet to attract and keep possession of some host organelles (mitochondria, the ER) by hijacking their molecular Velcro (Membrane Contact Sites), allowing the parasite to tap into the host power grid to fuel its own metabolic needs.

## Introduction

*Toxoplasma gondii* is a prime example of an obligate intracellular parasite that successfully co-opts host cell resources to enable development and, ultimately, persistence in the host. This parasite is able to invade almost any nucleated cell of its host by using its own motor system (consisting of unconventional myosin and actin filaments) for self-propelled entry, thereby bypassing host cell-mediated processes like phagocytosis [1]. This process of parasite-driven invasion involves the secretion of adhesive proteins from microneme secretory organelles, which enable the parasite to attach to the host cell. Among micronemal proteins, AMA1 localizes on the parasite surface post-secretion and is important for forming the moving junction, a tight ring-like contact point between the parasite and the host cell plasma membranes and serving as the entry portal [1]. AMA1 acts in concert with RON2, a protein secreted from the neck of the secretory organelle rhoptry, that is inserted into the host plasma membrane post-secretion and binds to AMA1, stabilizing the moving junction [1]. As the parasite progressively glides through the moving junction, this structure strips away host transmembrane proteins from the invaginated host cell plasma membrane [2,3]. Concomitantly, *Toxoplasma* secretes lipids and proteins (ROPs) from the rhoptry body into the host cell for incorporation into the nascent parasitophorous vacuole (PV) membrane (PVM) [4]. In addition to serving as a mechanical anchor, the moving junction functions as a molecular sieve, allowing the parasite to establish itself in a safe, self-made PV devoid of host-harmful proteins, i.e., recognition markers for lysosomal detection, fusion and destruction [2,3]. Once enclosed within the PV, the parasite secretes a diverse array of proteins, predominantly from the secretory dense granules (GRAs) that remodel the PV to interface with the host cell for promoting nutrient acquisition or thwarting immune destruction [5–8].

Over the past two decades, the remarkable extent and complexity of the *Toxoplasma* PV remodeling have become increasingly apparent. The PVM is no longer considered a simple passive barrier but instead is a highly dynamic and complex interface between the parasite and host cell. Several breakthroughs highlight that the parasite uses the PVM to selectively export effectors to modulate host cell pathways, subvert host immunity and hijack host cell resources [8,9]. *Toxoplasma* has shed entire metabolic pathways during its evolutionary path as an obligate intracellular pathogen, making host exploitation mandatory to obtain essential metabolites (lipids, purines, polyamines) for survival (reviewed in [10]). Its extreme genomic streamlining has forced this parasite to expand families of genes dedicated to nutrient scavenging, transport and processing [10]. In this Pearl, we review specialized mechanisms developed by *Toxoplasma* to create ‘scavenging portals’ in the PVM to scavenge various host resources. We focus on the proliferative tachyzoite form of the parasite as these phenomena have been characterized primarily in this life-cycle stage.

### 1. *Toxoplasma* constructs molecular sieve-like pores inserted into the PVM

As *Toxoplasma* dictates which components from the host plasma membrane can be incorporated into the forming PVM (allowing host lipids and GPI-anchored proteins and excluding host transmembrane proteins), it is very likely that the PVM does not contain any host bidirectional passive channels that would supply the parasite with small molecules or ions. Yet, studies using various membrane-impermeant dyes microinjected into *Toxoplasma*-infected cells demonstrated that the PVM is, in fact, permeable and permits the diffusion of charged and uncharged dye molecules up to 1,900 Da [11]. It has been further shown that PVM permeability is energy- and temperature-independent and not affected by active transport blockers, pointing toward the presence of parasite-derived, functional pores embedded into the PVM [11].

**Fig 1:**
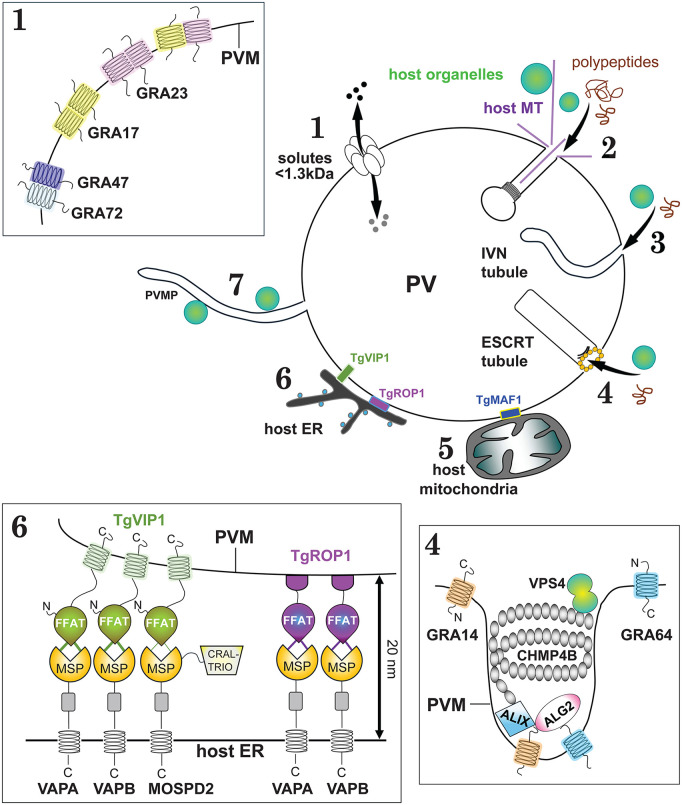
Schematic illustrations of a PV showing different types of *Toxoplasma* tachyzoite PV-host cell interactions. Multiple interactions occur between the PV and the host cell. (**1**) Pores inserted into the PVM, for small soluble metabolite uptake, composed of dimers of GRA17 (yellow), GRA23 (pink), GRA47 (dark blue) and GRA72 (light blue) (inset 1). Shown are homodimers and heterodimers based on co-immunoprecipitation data; the topology of the N- and C-termini of these GRAs has not yet been determined. (**2-4**) Different types of invaginations are formed at the PVM and allow host organelle, vesicle and protein internalization. In (**2)**: invaginations can be mediated by host microtubules (MT) poking into the PVM, (**3**) IVN tubules attached to the PVM and (**4**) inward buds created by host ESCRT proteins recruited at the PVM. Host ESCRT components (ALIX, ALG-2, CHMP4B, and VPS4) are recruited to the PV, mediated by the parasite effectors TgGRA14 and TgGRA64 (inset 4). The topology of TgGRA14 and TgGRA64 has been experimentally determined. (**5** and **6**). The close association of host organelles with the PVM. In (**5**): host mitochondria-PV contacts facilitated by TgMAF1, and (**6**) host ER-PV MCS mediated by TgROP1 (purple) and TgVIP1 (green) via their FFAT-like motifs with the MSP domains of host VAP family proteins (VAPA, VAPB, and MOSPD2). The predicted topology of TgVIP1 is shown. (**7**) PVM projections extend into the host cytosol for additional host organelle contacts.

Among the pore-forming proteins encoded by *Toxoplasma*, four GRA proteins localized at the PVM following secretion, have been identified so far, based on their ability to regulate PVM permeability (reviewed in [12]) (Fig 1, sketch 1). A first candidate is GRA17 that distributes as large foci at the PVM; AlphaFold-multimer structural predictions are consistent with a pore-like conformation, featuring a long N-terminal alpha helix that may function in channel closure and a C-terminal alpha-helical domain that forms a higher-order ring structure [13 15]. GRA17 contributes to the delivery of small molecules into the PV based on studies showing that most Δ*gra17* PV are impermeant to dyes, such as carboxy-dichlorofluorescein (445 Da) or Lucifer Yellow (522 Da) microinjected into infected cells [13]. Remarkably, the PV of Δ*gra17* parasites have an aberrant, enlarged morphology, referred to as ‘bubble vacuole phenotype’, with some of the vacuoles bursting or collapsing [13]. Consequently, Δ*gra17* parasites have reduced growth and virulence, potentially due to PVM pore malfunction, leading to parasite starvation by nutrient restriction and/or the accumulation of toxic metabolic byproducts inside the PV [13,14].

GRA23 is the second candidate pore protein that distributes all around the PVM [13]. Although *gra23* deletion does not cause any noticeable phenotype, GRA23 exhibits pore activity similar to GRA17, in assays using *Xenopus* oocytes as a heterologous expression system to study ion channels [13]. When individually expressed in oocysts, GRA17 and GRA23 localize to the plasma membrane and, in the presence of water, alter membrane potential sufficiently to induce oocyte cytolysis, which is consistent with the formation of non-selective pores [13]. Interestingly, GRA23 expression levels are increased by 1.8-fold in the Δ*gra17* strain, compared with wild-type parasites, suggesting that GRA17 and GRA23 may have compensatory functions, operating either independently as monomers on the PVM or assembled into multimers that function cooperatively as a channel complex [13]. Notably, GRA17 and GRA23 are synthetically lethal in *Toxoplasma*, indicating that the parasite needs at least one functional pore-type protein to survive [13,15].

**Fig 2:**
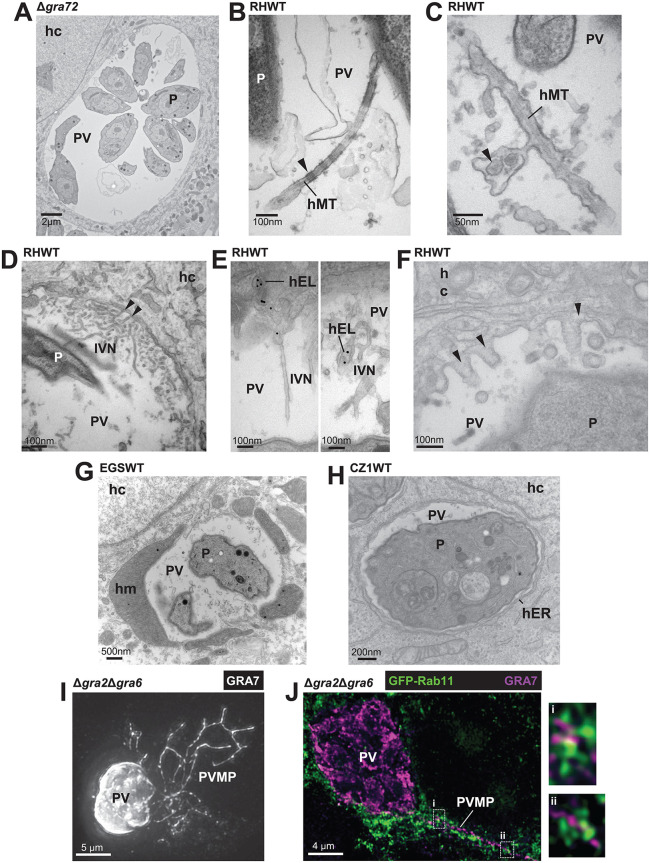
Morphological properties of the *Toxoplasma* PVM. Microscopy evidence of the PVM interactions schematized in Fig 1. **(A)** EM of a Δ*gra72* PV showing the ‘bubble phenotype’; **(B** and **C)** EM of a wild-type PV from the RH strain (RHWT) illustrating host microtubule (hMT)-based PVM invaginations, covered with a electron-dense material made of proteins (arrowhead in **B**), and containing host vesicles (arrowhead in **C**); **(D** and **E)** EM of RHWT PV showing IVN tubules appended to the PVM (arrowheads in **D**), acting as entry gates for host organelles, e.g., endo-lysosomes (hEL) containing LDL-gold shown in **E**; **(F)** EM of DN CHMP4B-based invaginations of a RHWT PV, revealing spiral filaments (arrowheads); **(G)** EM of host mitochondria (hm) attached to the PVM of wild-type parasites of the Type I/III EGS strain (CZ1WT) in cardiomyocytes (P, parasite); **(H)** EM of host host ER (hER) attached to the PVM of wild-type parasites of the Type II CZ1 Strain (CZ1WT) in fibroblasts (hc, host cell); **(I)** Immunofluorescence image showing the PVM and its projections, detected using anti-GRA7 antibody, of Δ*gra2*Δ*gra6* parasites (RH strain), and **(J)** VERO cells expressing GFP-Rab11A (green) infected with Δ*gra2*Δ*gra6* parasites, with the PVM and PVMP detected with anti-GRA7 antibodies (magenta). Host GFP-Rab11A vesicles associate with the PVMP (insets i and ii).

Interestingly, Δ*gra17* parasites adapt to in vitro culture after several passages, forming less swollen PV and showing faster growth [15]. In addition to *gra23*, approximately 80 other genes are upregulated at least 1.5-fold in the ‘nutrient sensitized’ Δ*gra17* mutant, compared with the wild-type parasites. A genome-wide synthetic lethality screen identified a gene encoding for GRA72 as genetically lethal in combination with the *gra17* gene. As the third candidate pore protein, GRA72 is a PVM transmembrane protein that shares predicted structural similarities with GRA17 for a pore-like structure formation. Furthermore, deletion of *gra72* results in the formation of ‘bubble PV’ (Fig 2, panel A) and reduced PVM permeability to small molecules.

Co-immunoprecipitation assays of infected cell lysates identified GRA47 as a binding partner of GRA72 [16,17] (Fig 1, inset 1). Localized to the PVM, GRA47 is the fourth candidate pore protein as its deletion results in the formation of ‘bubble PV’ and decreased PVM permeability, similar to phenotypes observed for Δ*gra17* and Δ*gra72* PV. Structural predictions indicate that GRA47 and GRA72 assemble into heptameric and hexameric pores, respectively, with conserved histidine residues lining the pore, and mutational analysis highlights the critical role of these histidines for protein functionality [15,17]. The role of GRA47 and GRA72 as pore-forming proteins was confirmed in the *Xenopus* oocyte system, showing that similar to GRA17, GRA47 and GRA72 alter the ionic currents and resting membrane potential of oocytes, consistent with pore formation [17]. As observed for Δ*gra23*Δ*gra17* and Δ*gra17*Δ*gra72* mutants, the double knockout strains Δ*gra17*Δ*gra47*, Δ*gra23*Δg*ra47* and Δ*gra72*Δ*gra47* are not viable [16]. Although GRA17, GRA47 and GRA72 each, complement their respective knockout strains, overexpression of any one of the other proteins (GRA17, GRA23, GRA47 or GRA72) in the Δ*gra17*, Δ*gra47* or Δ*gra72* strains do not fully rescue the ‘bubble PV’ phenotype, suggesting partial functional redundancy of these proteins [16].

GRA17, GRA47 and GRA72 are all required for *Toxoplasma* pathogenicity in vivo [13,15,17]. Overall, the permeability properties of the PVM enable the parasite to access host cytosolic essential nutrients, such as amino acids, monosaccharides and nucleotides.

### 2. *Toxoplasma* remodels the PVM to create invaginations as entry gates into the PV

Helium ion microscopy studies performed on *Toxoplasma*-infected cells to capture PVM surface topologies in 3-D at subnanometer resolution revealed the presence of several pore-like structures on the inner surface of the PVM, including tiny, smooth structures (~100 nm-long and ~60 nm-wide) scattered homogenously along the membrane surface, and circular pore-like openings of varying diameters (<10 nm, ~ 100–200 nm and 300 nm) [18]. Although the mechanisms underlying the formation and function of these varied openings remain unknown, their presence hints that the PVM has a highly dynamic and sophisticated structural organization. So far, three types of PVM invaginations created by *Toxoplasma* have been identified and partially characterized, and further evidence shows that these structures enable the parasite to access host macromolecules, lipophilic metabolites, polypeptides and even organelles, that cannot obviously pass through the pore complexes described above.

First, *Toxoplasma* actively remodels the host cell microtubule network by hijacking the microtubule organizing center (MTOC/centrosome), detaching it from the nucleus for relocation at the surface of the PV [19–21]. Consequently, the PV becomes encased by host microtubules, some of which protrude into the PVM to form deep invaginations extending up to 1.2-µm into the PV lumen, as illustrated by EM [19] (Fig 1, sketch 2). Ultrastructural observations of these microtubule-based invaginations also revealed several regularly spaced, externally coated striations (~25 nm thick made of proteins), associated with local membrane constrictions (Fig 2, panel B). Their role in the uptake of host cell-derived materials has been demonstrated using exogenous endocytic tracers (e.g., LDL-gold particles) to track the distribution of host endocytic organelles relative to the PV [19]. These studies showed that host endo-lysosomes accumulate at the tips in the microtubule-based invaginations (Fig 2, panel C). These structures named H.O.S.T. (Host Organelle-Sequestering Tubulo-structures) are stabilized by the dense granule protein GRA7 [19]. Together, these findings indicate that the parasite manipulates the host endo-lysosomal trafficking pathway via the recruitment of host microtubules to acquire nutrient-rich organelles.

Second, *Toxoplasma* generates membrane tubules (up to 60 nm in diameter) that are initially packaged within an apical membrane-bound compartment and subsequently expelled from the basal end of the parasite into the PV [22,23]. Once released inside the PV, these tubules spread throughout the lumen to form a complex intravacuolar network (IVN) that occupies a large portion of the vacuolar space, and some IVN tubules are seen appended to the PVM (Fig 2, sketch 3). These tubules are primarily formed by the dense granule proteins GRA2 and GRA6 via their amphipathic alpha-helices that insert into a lipid bilayer [24], which drives membrane tubulation [25]. Loss of either protein affects IVN biogenesis, resulting instead in the accumulation of small, sparse vesicles within the mutant PV [24]. Besides GRA2 and GRA6, other proteins are further incorporated into the IVN, ranging from structural proteins (e.g., GRA4, GRA12) to enzymes (e.g., kinases, phosphatases, lipases) [6]. A PV-resident kinase WNG1 phosphorylates at least ten GRA proteins, including GRA2, GRA6 and GRA7, which promotes their trafficking to the IVN for membrane association [26]. PV lacking *WNG1* show disrupted IVN membranes, revealing the importance of WNG1 in IVN stabilization. Scanning and transmission EM illustrate many IVN tubules appending to the PVM as a result of fusion events [22,27] (Fig 2, panel D), creating open conduits for the capture of host-derived materials into the PV, such as host endo-lysosomes and Rab vesicles [28,29] (Fig 2, panel E). Mutant parasites lacking an IVN internalize fewer host cytosolic proteins, Rab vesicles and organelles, with reductions up to 80% compared with wild-type parasites, highlighting the importance of this uptake pathway [28,30]. IVN tubules sequestering host organelles subsequently accumulate at the center of PV containing greater than 4 parasites, indicating detachment from the PVM [28].

Third, *Toxoplasma* secretes GRA proteins that localize at the PVM and harbor sequence motifs for interaction with host components of the Endosomal Sorting Complexes Required for Transport (ESCRT) machinery. Among diverse roles, the ESCRT pathway is a key mediator of multivesicular body biogenesis, driving the inward budding of endosomal membrane to form intraluminal vesicles (reviewed in [31,32]). Proximity labeling studies using a PVM-targeted probe combined with proteomic analyses of PVM-localized parasite proteins with ESCRT-interaction motifs identified many host ESCRT proteins facing the PVM: TSG101, VSP28, VPS37A, VPS27C from the ESCRT-I complex (which captures ubiquitinated transmembrane cargo and bridges ESCRT-0 and ESCRT-II complexes on endosomal membranes to initiate membrane budding) and CHMP1A, CHMP4A, CHMP4B, VPS4 from the ESCRT-III complex (which assembles into spiral filaments that bend and drives membrane scission), in addition to several ESCRT-accessory proteins such as CC2D1A, ALG-2/PDCD6, ALIX/PDCD6IP, PEF1, UBAP1 and UMAD1 [33–36]. Among parasite proteins harboring ESCRT-interaction motifs, GRA14 contains PTAP and YPX(n)L motifs for TSG101 and ALIX interaction, respectively, both located at its C-terminus that is exposed to the host cytosol [35]. GRA64 has its N-terminus facing the host cytosol and containing the YPRKQSTHL motif for ALIX interaction [36]. Following the recruitment of ESCRT proteins at the PVM mediated by these parasite proteins, this membrane undergoes deformations with inward protrusions, followed by bud fission, analogous in several ways to multivesicular body remodeling [37] (Fig 1, sketch and inset 4). Some IVN tubules are observed attached to ESCRT-mediated invaginations, suggesting cooperativity between the IVN and ESCRT-mediated pathways for host resource acquisition. Dominant-negative (DN) ESCRT-III mutants are commonly generated by fusing bulky tags to the C-terminus of CHMP proteins, causing them to remain in an open conformation and preventing ESCRT-mediated membrane scission with the AAA + ATPase VPS4 that powers the disassembly of the ESCRT-III protein complex [38–40]. Ectopic expression of DN CHMP4B-mEmerald in mammalian cells results in the blockade of the multivesicular body formation and, in *Toxoplasma*-infected cells, it leads to CHMP4B-mEmerald accumulation at the PVM [37]. Consequently, the PVM undergoes long tubular invaginations (up to 1.5 µm), enclosing CHMP4B filaments that further sever the lipid bilayer to a regular diameter of 110 nm (Fig 2, panel F). Upon DN CHMP4B-mEmerald expression, the PVM of Δ*gra14* and Δ*gra64* parasites forms spare and small buds positive for CHMP4B [37]. The DN mutant of VPS4 (VPS4A^EQ^), which is defective in ATP hydrolysis, prevents ESCRT-III disassembly [41]. Ectopic expression of DN VPS4A^EQ^ in *Toxoplasma*-infected cells results in failed scission of PVM tubules, leading to the accumulation of host organelles in large, membrane-bound structures still attached to the PVM [37]. Compared to wild-type parasites, the Δ*gra14* or Δ*gra64* strains have less recruitment of host ESCRT components to the PVM and less internalization of host proteins and Rab vesicles inside the PV, compatible with an impairment in PVM invagination formation [35,37]. This suggests that ESCRT-based invaginations significantly contribute to the delivery of host material to the PV. However, Δ*gra14* and Δ*gra64* parasites have normal growth, pointing to the role of additional proteins than GRA14 and GRA64, capable of interacting with host ESCRT proteins at the PVM [35,36].

### 3. *Toxoplasma* builds specialized contact sites with host organelles tethered to the PVM

Shortly after invasion, *Toxoplasma* recruits host mitochondria and ER at the PVM, where it forms close and sustained interactions with these organelles until egress from the host cell [42–46] (Fig 1, sketches 5 and 6). Recruited host mitochondria align parallel to the PV, forming areas of close membrane apposition between the outer mitochondrial membrane and the PVM, at intermembrane distance averaging 12–20 nm (Fig 2, panel G)**.** Host mitochondrial association is not a universal feature of all *Toxoplasma* strains: virulent Type I and avirulent III strains actively recruit and tether host mitochondria to the PVM whereas the cystogenic Type II strains survive without interacting with these organelles [44]. The wrapping of host mitochondria around the PV is driven by TgMAF1b, a PVM-located GRA protein expressed in Type I (RH, GT1) and Type III (VEG, CTG) strains, but not in Type II strains (ME49, PRU) [44]. The *T. gondii* genome contains 6–10 copies of the MAF1 gene due to expansion of the MAF1 locus [47]. Type II strains instead express the paralog TgMAF1a, which shares 60% sequence identity with TgMAF1b and localizes to the PVM without host mitochondria recruitment. Deletion of *MAF1b* in Type I strains results in the abrogation of mitochondrial tethering at the PVM but does not result in a significant growth disadvantage in cultured cells [44]. Mice infected with type I Δ*maf1* parasites succumb to infection at rates comparables to those of mice infected with wild-type parasites but exhibit different cytokine profiles, suggesting a link between host mitochondrion association with the PVM and innate immune signaling [44]. Interestingly, ectopic expression of TgMAF1b in Type II strains confers a competitive growth advantage over wild-type parasites [47].

The host binding partners of TgMAF1b required for host mitochondrial association include the outer mitochondrial membrane import receptor TOM70 and the chaperone HSPA9, both involved in cellular stress responses [48]. Δ*MAF1b* parasites cannot replicate in host cells deficient for TOM70 and this requirement may relate to the role of TOM70 as a stress response gatekeeper, preventing toxic protein aggregation and mitochondrial damage. In fact, TgMAF1b interaction with TOM70 induces the shedding of damaged outer mitochondrial membrane structures to clear localized stress, likely caused by the infection, while leaving healthy parts of the mitochondria for ATP production [49]. In addition to MAF1b, the rhoptry kinase protein ROP39 localizes to the PVM of the Type I RH strain, with partial colocalization with host mitochondria, and this protein may be involved in the recruitment of these organelles via its kinase activity as Δ*rop39* parasites show a 10% decrease in host mitochondrial association with the PVM [50]. Host mitochondria may serve as a source of ATP, fatty acids, folate, lipoate or choline for the parasite [29,51–53].

Like host mitochondria, ER tubules are tethered to the PVM at close distances ranging from 8 to 25 nm [45,46]. All *Toxoplasma* strains recruit host ER, with higher coverage for PV of Type II strains (up to 63% for ME49 and 96% for CZ1) [45,46] (Fig 2, panel H). The PV is functionally wired into the host ER through the establishment of membrane contact sites (MCS), specialized interfaces where the limiting membranes of two organelles come into close proximity (typically ~10–50 nm apart) without fusing [54]. In mammalian cells, MCS serve as structural platforms that govern several cellular processes, including the non-vesicular lipid transfer mediated by Lipid Transfer Proteins (LTP, e.g., oxysterol-binding proteins, phosphatidylinositol transfer proteins, ceramide transporter) and intracellular signaling through calcium and metabolite exchange [54,55]. The ER MCS machinery relies on specific protein tethers, such as the highly conserved VAMP-associated proteins (VAPs) [56]. These proteins exposed their N-terminal MSP domain to the cytosol for interaction with cytosolic or organellar proteins containing a conserved FFAT motif (two phenylalanines in an acidic tract), or related motifs like phospho-FFAT [56]. Three host VAP proteins (VAPA, VAPB and MOSPD2) localize at the PVM [45,46,57], and in mammalian cells deficient in all these proteins, no host ER is recruited at the PV, severely impairing *Toxoplasma* growth [46]. So far, two parasite proteins have been identified as partner proteins for host VAPs: at the onset of infection, TgROP1 binds to VAPA via the DDTFHDALQE motif [45] and during parasite replication, the dense granule protein TgVIP1 binds to VAPA, VAPB and to a lesser extent MOSPD2 via the TFFDALE motif [46] (Fig 1, inset 6). Deletion of *rop1* or *vip1* results in reduced ER coverage of the PV surface [45,46]. Parasites lacking *vip1* replicate poorly in vitro and abnormally accumulate acidocalcisomes, organelles involved in calcium and phosphorus storage [46]. These defects are more pronounced in host cells lacking VAPA,VAPB and MOSPD2, suggesting a potential role of host ER recruitment in calcium exchange with the parasite.

Jointly, these observations highlight that ER-PV MCS establishment is under strong selective pressure, representing a foundational feature of the intracellular niche of *Toxoplasma*, with a potential therapeutic vulnerability. *Toxoplasma* deploys distinct yet coordinated secretory programs to reroute host mitochondria and ER to its vacuole, promoting close association with the PVM, and thereby creating an integrated metabolic and signaling microenvironment to support its survival.

### 4. *Toxoplasma* forms tubular projections emerging from the PVM for host cell interactions

A hallmark of the intracellular parasitism of *Toxoplasma* is the expansion of the surface area of the PV via the formation of long membrane tubules that extend into the host cytoplasm (Fig 1, sketch 7). These projections that bud from the PVM appear early during infection as short tubules that can elongate to reach lengths up to 50 µm; however, only few PVM projections persist during replication [28]. Following host cell invasion, some PVM projections extend towards the host MTOC, which becomes delocalized from the nucleus to the PV [19–21]. PVM projections contain several GRA proteins including GRA3, GRA7 and GRA14 [21,58,59]. Projections connecting two PV can facilitate the exchange of materials or proteins between PV, as demonstrated for GRA14 [59]. Interestingly, IVN-deficient parasites, which are impaired in the internalization of host endo-lysosomes and Rab vesicles yet have no growth defects, form numerous PVM projections that permeate the host cytoplasm and are maintained throughout infection until egress [28] (Fig 2, panel I). This phenotype may represent a compensatory adaptation to the lack of IVN, whereby outward extensions of the PVM provide extra surface area and enhance host organellar contacts such as Rab11 vesicles for molecular delivery of nutrients (Fig 2, panel J).

### 5. Once nutrients reach the PV lumen, *Toxoplasma* organizes their trafficking and processing

*Toxoplasma* installs many mechanisms at the PVM to acquire host cell-derived metabolites, including pore-mediated diffusion, PVM invaginations-mediated transport and host MCS hijacking. Once delivered into the PV, nutrients then need to be available to the parasite. While small soluble cytosolic molecules capable of passing through PVM pores (such as glucose, purines, amino acids), can freely diffuse within the PV and subsequently be imported into the parasite by specific plasma membrane transporters, lipophilic metabolites and nutrients conveyed by host organelles would need to be processed in the PV before being available to the parasite. *Toxoplasma* has many metabolic deficiencies or production limitations for lipids [10]. For example, the parasite is unable to synthesize cholesterol de novo [19], cannot produce sufficient amounts of phosphatidylcholine, phosphatidylserine, phosphatidylethanolamine or phosphatidylinositol species to support its fast growth [60] and despite its capability to synthesize sphingolipids, it also salvages these lipids from the host cell [28,61–64]. Current evidence indicates that the parasite exploits various host cell-derived lipid sources. Cholesterol is acquired from plasma LDL endocytosed by the host cell and conveyed to the PV via endo-lysosomes and endocytic Rab vesicles [19,28]. Host Golgi-derived sphingolipids are delivered to the PV via Rab14-, Rab30- and Rab43-positive vesicular pathways. The source/s of host phospholipids is still unresolved but could be the host ER and/or mitochondria attached at the PVM, potentially through transfer mediated by MCS.

*Toxoplasma* secretes a GRA protein with a lecithin:cholesterol acyltransferase activity, TgLCAT that localizes on the IVN [65]. Like any LCAT, TgLCAT converts phosphatidylcholine into lysophosphatidylcholine, releasing a free fatty acid that esterifies a molecule of cholesterol. Because lysophosphatidylcholine has an inverted cone-shaped molecular geometry, this lipid can disrupt and solubilize cell membranes. IVN membranes positive for TgLCAT are frequently observed surrounding intra-PV host organelles and Rab vesicles; in parasites overexpressing LCAT, fewer host Rab vesicles are detected inside the PV, suggesting that TgLCAT functions as a membrane-damaging enzyme and permeabilizes the membrane of internalized host organelles to promote the release of their content [28]. To prevent damage to its own membranes, *Toxoplasma* regulates TgLCAT lipolytic activities by synthesizing the enzyme as an inactive precursor that is post-translationally cleaved into two fragments that are reassembled into a functional enzyme on the IVN [65]. Of note, the parasite secretes a GRA protein acting as an inhibitor of serine proteases TgPI-1, that localizes to IVN tubules [66]. Although the role of TgPI-1 in the PV is still unknown, this protein may protect the parasite from host lysosomal serine proteases (cathepsin A, cathepsin G) liberated following the disruption of host lysosomal membranes inside the PV.

Lipids released in the PV lumen can be transported by a GRA protein belonging to the SEC14/CRAL-TRIO superfamily [67]. Members of this family harbor a hydrophobic ligand-binding cavity along with a flexible gate-like structure that regulates lipid entry and exit [68,69]. This pocket can accommodate a single lipid molecule, such as a phosphoinositide, phosphatidylcholine, diacylglycerol or sterol. In *Toxoplasma*, the protein TgSEC14-LTP1 localizes to the PV including the IVN, and facilitates the transport of host-derived phosphatidylcholine and diacylglycerol [67]. Depletion of TgSEC14-LTP1 impacts parasite lipid homeostasis, by disrupting phosphatidylcholine pools and lipid droplet biogenesis, ultimately impairing parasite growth [67].

These findings support a central role for the IVN as a hub in promoting lipid availability and trafficking to feed the parasite. From a therapeutic perspective, the TgSEC14-LTP1 lipid binding pocket may be druggable, as previously demonstrated for the SEC14 proteins in the pathogenic fungi *Cryptococcus neoformans* and *Candida albicans* in which small molecules disrupt ligand occupancy and effectively block the lipid-dependent functions of these proteins [70].

### 6. Does host–PV interactions in *Toxoplasma* share a fundamental blueprint with other Apicomplexa?

The phylum Apicomplexa encompasses several thousand species that show remarkable diversity in their complex life cycles, with morphologically distinct life stages and one or more hosts. Despite evolutionary divergence that has led to structurally distinct of PVM containing specific protein sets, intravacuolar Apicomplexa employ the same core mechanisms to modify their PV and acquire host cell-derived nutrients.

- ***The malaria parasite:*** *Plasmodium falciparum* infecting erythrocytes forms a permeable channel in the PVM that permits the diffusion of solutes up to ~1,400 Da from the host cytosol and it transports amino acids and monosaccharides across the PVM [71–73]. The GRA protein EXP2 is the primary core component of the channel, forming homo-oligomers that assemble into a heptameric pore [74–76]. PfEXP2 shares AlphaFold structural predictions with *Toxoplasma* GRA17 and GRA72 [15,76]. Expression of PfEXP2 in ∆*gra17*, ∆*gra47* or ∆*gra72* restores the ‘bubble’ phenotype of these *Toxoplasma* mutant parasites [13,16], indicating analogous functions in nutrient import via a pore. Identified by immunoprecipitations of Pf EXP2, PfEXP1 is required for proper organization of PfEXP2 in the PVM [77]. *EXP2* knockdown in *P. falciparum* is detrimental for parasite growth [75], and the nutrient-permeable channel activity is defective in Δ*EXP1* parasites [78]. In hepatocytes, *Plasmodium berghei* modifies the PVM permeability, allowing the passage of solutes up to 855 Da, suggesting the presence of pores of unknown composition [79]. To date, neither an IVN nor PVM invaginations equivalent to those observed in the *Toxoplasma* PV have been described in the PV of *Plasmodium* spp. However, the PV of erythrocytic (*P. falciparum*) and hepatic (*P. berghei* and *Plasmodium vivax*) parasites form long tubular extensions of the PVM in the host, forming a tubulovesicular network (TVN) [80–82], reminiscent of the PVMP of *Toxoplasma*. The *Plasmodium* TVN contains GRAs, including EXP1, and interacts with host late endosomes, lysosomes and autophagic organelles in hepatocytes [83–85]. Some regions of the PVM of intraerythrocytic *P. falciparum* are closely apposed to the parasite plasma membrane (~10 nm) [86] in which the Niemann-Pick type C1-related protein PfNCR1 is localized, suggesting a potential transfer of lipids to the parasite at these zones of contact [87]. *P. berghei* liver stage parasites recruit host ER for physical contacts with the PVM [79], suggesting potential MCS. Host Golgi vesicles and late endosomes (e.g., Rab7A vesicles) gather around the PV and the content of endocytic organelles is observed in the PV lumen, but the mechanism of PVM crossing is undetermined [88,89].
- ***The agent of neosporosis Neospora caninum:*** This parasite infects enterocytes and cells from the brain, muscle and placental cells causing abortion in cattle. *N. caninum* shares with T. gondii a core ancestral genome and many host cell remodeling features, such as the reorganization of the host microtubule cytoskeleton, close association of the PVM with host mitochondria, attraction of host lysosomes (sources of cholesterol), multivesicular bodies and Golgi vesicles to the PV, and internalization of host Rab14 vesicles (sources of sphingolipids) into the PV [90].

## Outstanding questions for future directions and implications

While research on the nutritional needs and salvage pathways developed by *Toxoplasma* tachyzoites has advanced significantly this last decade, there are still fundamental knowledge gaps and priorities for future exploration should include a better understanding host cell-parasite metabolic cross-talks and examination how these unique salvage pathways can be targeted for novel therapeutic development [12,91].

- Are there specialized PVM subdomains?
- In pore-deficient mutants, what causes the formation of swollen, distorted ‘bubble PV’?
- Which parasite proteins interact with host microtubules to mediate PVM invaginations?
- Which parasite proteins at the PVM selectively recognize host organelles for selective PV entry?
- Is there a suborganization of IVN tubules?
- How are scavenged host-derived lipids, such as cholesterol, transferred from the PV to the parasite?
- Besides nutrient salvage, are host ESCRT recruited at the PVM involved in other functions like membrane injury repair?
- How is the transition from rhoptry-initiated to dense granule-reinforced ER-PV contacts synchronized?
- Which metabolites traverse ER-PV MCS to benefit the parasite?
- How do PVM projections form, grow and extend?
- What are the lipid sources and protein composition of the PVM projections?
- Do PVM projections form functional MCS with host organelles?

Notably, *Toxoplasma gondii* needs to adjust its nutrient acquisition strategies depending on its lifecycle phase, shifting from proliferative stage (tachyzoites) with a high demand for scavenging systems for high-volume supply of nutrients to a slow-growing, less metabolically active cyst form encapsulated in a thick cyst wall. While this review focuses on the nutrient needs and uptake pathways developed by tachyzoites, much more has to be discovered on the metabolic adaptability and selective nutrient transport systems of the parasite during the chronic persistence in the host.

Finally, it is worth mentioning that, in the context of host–pathogen interactions, while pathogens reprogram host metabolism and extract nutrients, host cells have developed countermeasures in this battle for metabolites [92–95]. For example, infected cells have evolved mechanisms to restrict access to crucial metabolites such as essential amino acids, folate, and fatty acids to limit *Toxoplasma* growth [96,97]. In addition, *Toxoplasma* disrupts host ER homeostasis and induces ER-stress, to which host cells respond by activating the unfolded protein response (UPR) and ERphagy [98]. These host degradation pathways are further exploited by the parasite to secure amino acids and lipids for its own growth and persistence [98]. These findings may inspire the development of new antiparasitic therapies.
